# Patient, Surgery, and Hospital Related Risk Factors for Surgical Site Infections following Total Hip Arthroplasty

**DOI:** 10.1155/2015/979560

**Published:** 2015-05-14

**Authors:** Georgios Triantafyllopoulos, Ottokar Stundner, Stavros Memtsoudis, Lazaros A. Poultsides

**Affiliations:** ^1^Department of Orthopaedic Surgery, Division of Adult Reconstruction and Joint Replacement, Hospital for Special Surgery, Weill Medical College of Cornell University, 535 East 70th Street, New York, NY 10021, USA; ^2^Department of Anesthesiology, Perioperative Medicine and Intensive Care Medicine, Paracelsus Medical University, Muellner Hauptstrasse 48, 5020 Salzburg, Austria; ^3^Department of Anesthesiology, Hospital for Special Surgery, Weill Medical College of Cornell University, 535 East 70th Street, New York, NY 10021, USA

## Abstract

Surgical site infections (SSI) following total hip arthroplasty (THA) have a significantly adverse impact on patient outcomes and pose a great challenge to the treating surgeon. Therefore, timely recognition of those patients at risk for this complication is very important, as it allows for adopting measures to reduce this risk. This review discusses literature reported risk factors for SSI after THA. These can be classified into patient-related factors (age, gender, obesity, comorbidities, history of infection, primary diagnosis, and socioeconomic profile), surgery-related factors (allogeneic blood transfusion, DVT prophylaxis and coagulopathy, duration of surgery, antibiotic prophylaxis, bearing surface and fixation, bilateral procedures, NNIS index score, and anesthesia type), and hospital-related factors (duration of hospitalization, institution and surgeon volume, and admission from a healthcare facility). All these factors are discussed with respect to potential measures that can be taken to reduce their effect and consequently the overall risk for infection.

## 1. Introduction

The success of total hip arthroplasty (THA) in relieving pain and improving function in patients with end-stage degenerative or inflammatory arthritis of the hip is undisputed. The number of procedures per year is estimated to further rise in the next years, as even younger patients with hip arthritis are expected to seek surgical treatment. Kurtz et al. [1] recently reported that, by the year 2020, the projected number of THAs per year will exceed 500,000. Periprosthetic joint infection (PJI) remains a devastating complication after THA, as its treatment may involve multiple surgical interventions and long-term administration of antibiotics and is associated with poor patient satisfaction and increased socioeconomic costs [2, 3]. PJI is also correlated with increased mortality, particularly in the group of elderly patients [4]. The rate of surgical site infection (SSI) has been reported to range from 0.2% during hospitalization to 1.1% up to 5 years after surgery [5]. Therefore, the projected increase in the number of THA will also reflect a respective increase in the absolute number of patients presenting with this complication.

Measures including perioperative antibiotics, exhaust suits, laminar air flow operating rooms, ultraviolet lighting, and antibiotic-impregnated cement have been introduced in an attempt to control infection rates after joint replacement surgery. However, before treating a patient with arthritis, it is very important that surgeons are able to identify potential risk factors for developing an infection. The purpose of this review is to summarize those risk factors predisposing to SSI following THA.

## 2. Search Strategy and Selection Criteria

An unrestricted computerized search of MEDLINE and OVID SP for studies published in the English language between 1990 and 2014 was conducted. The following terms were used in various combinations: “total hip arthroplasty,” “risk factors,” “infected joint arthroplasties,” “surgical site infection,” “periprosthetic joint infection,” “prosthesis-related infection,” “joint replacement infections,” “musculoskeletal wound infections,” and “osteomyelitis.” Abstracts were reviewed and studies reporting on risk factors for superficial or deep PJI following THA were selected for full text review.

## 3. Patient-Related Factors

### 3.1. Age and Gender

The risk for infection after THA has been found to be higher in older patients [6–8]. In comparison to the younger population, these patients are usually characterized by impaired immune response to infectious agents, inferior nutritional status, and possibly more comorbidities. On the other hand, other investigators have determined younger age as a risk factor for infection after total joint arthroplasty [9]. In another study [10], the same association between younger age and infection risk was found for total knee arthroplasty but not for THA. The higher odds for future revision surgery related with younger patients has been proposed as an explanation of the increased infection risk in this population [9].

Male gender has been also reported as an independent risk factor for infection after THA [10–13]. Higher activity levels increase the risk for subsequent revision surgery, and also gender differences in skin microbial colonization [13] might account for this effect. In contrast to this, other studies showed an association for increased infection risk with female gender [6, 14].

### 3.2. Obesity

As obesity is considered a contemporary pandemic and its association with degenerative joint disease is known [15], obese patients present very frequently for THA. Nonetheless, morbidly obese patients undergoing THA have been found to have significantly higher rates of complications compared to nonobese patients, within 5 years postoperatively [16]. A more specific correlation between obesity and a higher risk for SSI after THA has been reported by several investigators [7, 9, 14, 17–26]. It has been shown that a BMI ≥ 35 is associated with a higher risk for positive intraoperative cultures during THA [26]. Furthermore, morbid obesity has been correlated with prolonged wound drainage [27], which is a known risk factor for deep SSI [8, 28]. Treating morbid obesity before THA can be therefore beneficial to patients not only from a general health perspective, but also in terms of reducing the risk of developing a postprocedural SSI. Diet and lifestyle modifications, pharmaceutical interventions, psychological support, and even bariatric surgery can be considered in these patients. This requires a multidisciplinary approach, involving the collaboration between the treating surgeon and a clinical dietician, an internist, a psychiatrist, and a bariatric surgeon. However, even with these measures, it is very unlikely that an obese patient scheduled to undergo THA will achieve such a preoperative weight loss that can have an effect on the risk for infection. It appears that a substantial preoperative weight-loss before surgery is needed for a significant effect to take place [29].

### 3.3. Comorbidities

The American Society of Anesthesiologists (ASA) score reflects the impact of existing comorbidities on the patient's general health status. An ASA score equal to or more than 3 has been determined by some investigators as an independent risk factor for SSI after THA [6, 14, 30]. Other studies have found an association between an increased risk for infection and the presence of more than 2 comorbidities [20, 24, 31], with each additional comorbidity significantly increasing this risk [31]. A Charlson comorbidity index of more than 5 has been also determined as an independent risk factor for PJI [12].

It is well established that, in patients undergoing surgery, diabetes mellitus (DM) is associated with increased risk for complications and increased length of hospital stay [32]. In the perioperative period, optimal glycemic control can be a challenge, as surgical stress in addition to modifications of the patient's usual diet needs to be taken into account. In general, hyperglycemia may cause disruption of the host's physiologic response to a bacterial load [33]. Diabetes has been identified as an independent risk factor for developing SSI after total joint arthroplasty, including THA [7, 9, 24, 31, 34, 35]. Even in patients without a diagnosis of DM, a fasting blood glucose of >140 mg/dL has been associated with a 3-fold increase in the risk of infection [24]. HbA1c is used to provide an estimate of the average blood glucose concentrations for a period expanding to 3 months before testing [32, 36]. However, its importance in predicting the risk for infection after total joint arthroplasty has not been confirmed [35]. Recognition of those patients with uncontrolled DM or even with perioperative fluctuations of the blood glucose levels is thus very important. In addition to interventions aiming to reduce bacterial load (e.g., use of antibiotic-impregnated cement), close monitoring of patients' perioperative blood glucose levels, and control of hyperglycemia (e.g., insulin sliding scale) can help minimize the risk of infection.

Connective tissue disease has been also correlated with increased risk for PJI after THA [11, 19, 37]. This group of conditions, including rheumatoid arthritis (RA), systemic lupus erythematosus, and psoriatic arthritis, is associated with modulation of the patient's immune system, resulting in predisposition to infection. Patients with longer duration of RA are at increased risk [38]. Moreover, these patients often receive chronic immunosuppressive treatment. Chronic glucocorticoid treatment has been identified as a risk factor for infection after THA [8, 18]. Novel biologic agents (TNFa blockers) used in the treatment of many of these conditions are known to adversely affect the patient's ability to fight infection and their use has been identified as a risk factor for PJI following total joint arthroplasty [38, 39]. It has been reported that patients with RA that have never received TNFa blockers have lower rates of bacteremia than those patients under biologic treatment [39]. Interruption of TNFa blockers before surgery has been advocated [38, 40]; however, other studies have failed to prove that this measure actually decreases the infection risk [41].

The presence of malignancy has been confirmed to increase PJI risk [8, 10, 42]. It remains unclear, however, whether this correlation is due to the potential effects of malignancy on the immune response to infection per se or to the associated treatment such patients receive, which frequently consists of chronic administration of glucocorticoids and cytotoxic agents [42].

A number of other comorbidities have been also described as independent contributors to the infection risk after THA. These include preoperative anemia [19], liver disease [8, 10, 43], alcohol [7, 8] and intravenous drug abuse [8], previous myocardial infarction [24, 25], congestive heart failure [10, 24], postoperative atrial fibrillation [25], renal insufficiency [24, 25], fluid and electrolyte disorders [10], and pulmonary circulatory disease [10].

### 3.4. History of Infection and* Staphylococcus aureus* Colonization

It has been supported that infection involving the superficial layers of the surgical site is an independent risk factor for subsequent deep PJI [8, 18, 42]. Furthermore, increased drain output has been correlated with superficial infection and therefore with indirectly elevated risk for deep infection following THA [18, 27]. Prolonged wound drainage and other signs of superficial infection should alert the treating surgeon and prompt to further diagnostic testing and management [8]. It should be noted that, even though a direct association between the use of drains and the risk for SSI after THA has not been proven [13], it is recommended that drains should be removed in a timely fashion [44].

Patients with previous PJI of the same or different site have been recognized to be at increased risk for an ensuing infection [37, 45]. However, this effect is not attributed to the multiple number of artificial joints, but rather to contamination from surgery or to patients' poor general health status and immunocompromise [45].

Despite the fact that direct contamination of the wound during surgery seems to be the primary pathogenetic mechanism, especially with regard to* Staphylococcus aureus* and* Staphylococcus epidermidis*, bacteremia related with the presence of infection at sites other than joints may also lead to hematogenous seeding of a prosthetic joint and development of PJI in a considerable percentage of patients [46]. Patients with THA and infections of the skin [8], respiratory [8], or urinary tract [8, 25], as well as dental [47] and abdominal infections [8], were found to be at increased risk for PJI. The bacterial load seems to play a critical role in this effect [48]. Asymptomatic bacteriuria has been also determined as a risk factor for infection with gram negative bacteria following THA [49]. Therefore, an aggressive treatment protocol and close monitoring of patients undergoing THA and an infection at another site is warranted.

Colonization with* Staphylococcus aureus*, when combined with other variables including active tobacco use and BMI ≥ 30 kg/m^2^, has been identified as a risk factor for infection after THA [23]. Similarly, other investigators have found that colonization or prior infection with* Staphylococcus aureus* as remotely as 10 years before presentation increases the risk for SSI after total joint arthroplasty [50]. Moreover, patients colonized with* methicillin-resistant Staphylococcus aureus* (MRSA) are known to be at increased risk for SSI after elective orthopaedic surgery [51, 52]. For these patients, the use of vancomycin for perioperative prophylaxis is advocated, as it is related with reduced infection rates [53].

### 3.5. Primary Diagnosis

Patients subjected to THA for posttraumatic osteoarthritis have been found to be at increased risk for infection [8]. In addition, higher rates of PJI have been reported in patients that underwent THA for traumatic hip injury (e.g., hip fracture) [6, 11, 34]. The local effects of a traumatic injury (hematoma formation, tissue necrosis, etc.), as well as its systemic consequences, may account for this correlation [6]. Dislocation, associated with local trauma and often requiring multiple reoperations, is another risk factor reported [8]. Analysis of the data retrieved from the Nordic Arthroplasty Register Association revealed that avascular necrosis of the femoral head was also correlated with increased risk for PJI [11]. Finally, several studies have indicated revision surgery [23, 34, 37] or prior joint arthroplasty [42] as an independent risk factor for developing infection following THA.

### 3.6. Socioeconomic Factors

Lower socioeconomic status, as indicated with entitlement to public assistance for Medicare, has been correlated with the risk of PJI [12]. Moreover, minority race has been also determined as an independent risk factor for SSI [10]. An increased risk for infection following THA has been also described for patients living in rural areas [7]. Poor living conditions, comorbidities, failure to adequately follow medical instructions, and delay in seeking help may predispose all these patients to increased infection risk.

## 4. Surgery-Related Factors

### 4.1. Allogeneic Blood Transfusion

Several studies have pointed out allogeneic blood transfusion as an independent risk factor for SSI after total joint arthroplasty, including THA [17, 25, 54, 55]. Transfusion of allogeneic blood is considered to induce immunomodulation to the recipient, due to the presence of white blood cells (WBC) above a critical level [56]. However, even transfusion of WBC-filtered allogeneic blood has been found to independently increase the risk of infection after THA [54]. Preoperative autologous blood donation, regional anesthesia [57], and prevention of excessive intraoperative blood loss can reduce the need for allogeneic blood transfusion [58] and therefore the risk of developing SSI.

### 4.2. DVT Prophylaxis and Coagulopathy

It has been reported that patients receiving low molecular weight heparin (LMWH) for DVT prophylaxis have significantly more prolonged wound drainage [27]. This has been attributed to the faster onset of action of LMWH in comparison to Coumadin. Nonetheless, aggressive anticoagulation both with warfarin and with heparin has been identified as an independent risk factor for SSI [17, 59, 60]. Thus, DVT prophylactic medications should be closely monitored during the early postoperative period and any adverse events, including prolonged drainage and hematoma formation, should be recognized and addressed in a timely fashion. Coagulopathy has been also correlated with an increased SSI risk [10, 19]. Coagulopathy can be the result of a wide spectrum of disorders, including those inherited (e.g., hemophilia) and those acquired (e.g., liver disease). However, in most cases, appropriate pharmacologic interventions can maintain the coagulation mechanism on a level that may permit surgery while minimizing the risk for complications.

### 4.3. Prolonged Duration of Surgery

Several studies have identified prolonged operating time as an independent risk factor for PJI of the hip [6, 8, 12, 13, 23, 24, 30, 34, 37]. Procedures requiring more time usually relate to complex cases and may involve extensile exposures and considerable tissue damage [6]. Dealing with issues such as suboptimal surgeon training, inadequate preoperative planning, and substandard cooperation of operating room staff may lead to improvement of surgical times and therefore contribute to the decrease of the infection risk [23].

### 4.4. Antibiotic Prophylaxis and Skin Preparation

Antibiotic prophylaxis is a well-established measure to control postoperative infections. Failure to adhere to protocols of prophylactic antibiotic administration, both in terms of dosing and timing, has been associated with increased risk for PJI in patients undergoing THA [8, 30]. Additionally, for cemented implants, the use of plain cement (i.e., nonimpregnated with antibiotics) has been correlated with increased risk for revision due to infection [11]. Finally, although a recent international consensus meeting on periprosthetic joint infections did not acknowledge any differences between different agents [61] and in contrast to previous data [62], in one study skin preparation with chlorhexidine was found to be associated with increased rates of superficial wound infection when compared to povidone iodine [17].

### 4.5. Bearing Surface and Fixation Type

An increased infection risk has been linked with the use of metal-on-metal bearings in THA [63]. Metallosis-related local soft-tissue damage and increased rates of revision associated with this type of implants may be contributing factors. Moreover, in a study of arthroplasty register data, hybrid fixation was identified as a risk factor for revision of a THA due to infection [11]. However, the authors suggested that this effect might be due to confounding factors not recorded in the register.

### 4.6. Bilateral Procedures

Bilateral THA has been associated with an increased risk for infection in some studies [14, 25]. It is therefore recommended that bilateral procedures should be reserved for patients without major comorbidities [25]. Nonetheless, as other researchers have supported the safety of this treatment modality [64, 65], further investigation of the role of bilateral procedures is warranted.

### 4.7. National Nosocomial Infections Surveillance (NNIS) Index Score

The NNIS index score reflects the patient's general health (as related to the ASA score) but also takes into account the procedure's duration and the condition of the surgical wound. A NNIS index score equal to or more than 1 has been identified as an independent risk factor for PJI [42].

### 4.8. Anesthesia

In recent years, a number of publications have suggested that the choice of regional versus general anesthesia may have a profound influence on the general and possibly the local infection risk in orthopedic patients [57, 66, 67]. Possible mechanisms suggested by various authors are beneficial effects of neuraxial anesthesia on tissue perfusion, immune function, and blood loss. While the effect on short-term outcomes is increasingly well documented, long-term outcome data are rare at this time.

Other aspects of anesthesia may also play a role in preventing SSI after THA. For example, given the effectiveness of intraoperative high FiO_2_ in preventing SSI in the general surgery population [68], the value of implementing such strategies in the field of elective orthopaedic surgery, including THA, needs to be further clarified. Additionally, controlled epidural hypotension has been shown to significantly reduce blood loss and improve tissue perfusion through vasodilatation. As hematoma formation and need for blood transfusion have been linked to increased risk of infection, hypotension may actually be beneficial. Care has to be taken however to not equate hypotension with hypo perfusion, which may be more likely during episodes of hypovolemia due to blood loss or general anesthetic effects.

## 5. Hospital-Related Factors

### 5.1. Duration of Hospitalization

Patients undergoing THA were found to have an increased risk for developing PJI if their hospitalization was prolonged [25, 42]. Similarly, non-same day surgery has been identified as an independent risk factor for PJI [23]. Hence, it is suggested that patient admission for an elective procedure such as THA should be avoided prior to the day of surgery, as longer hospitalization predisposes patients to greater exposure to nosocomial bacteria [25].

### 5.2. Hospital and Surgeon Volume of Procedures

Higher infection rates have been associated with low institution volume of THA procedures [69, 70]. It is likely that high-volume institutions have organized SSI control departments and strictly adhere to measures for prevention and early detection of infections. Additionally, low surgeon volume is another variable identified as a risk factor for SSI after THA [70, 71]. The number of cases surgeons perform seems to be conversely related to the duration of surgery and therefore may have an impact to the infection risk as well.

### 5.3. Admission from a Healthcare Facility

Patients admitted for THA from a healthcare facility have been found to have increased risk for developing SSI [72]. These patients have generally inferior health status than home-residing patients and are likely to be more prone to infections.

## 6. Conclusions

Identification of risk factors for SSI in patients undergoing THA allows for implementing measures to tackle those variables that can be modified and therefore to reduce the relevant infection risk. These measures may include adequate perioperative glycemic control, adjustment of the DVT prophylactic regimen to the needs of each individual patient and close monitoring of the coagulation status, increased awareness for early signs of superficial infection, both surgeon and patient education, stringent protocols of perioperative antimicrobial prophylaxis, and so forth. It should be noted that most studies reporting on the aforementioned risk factors are of retrospective nature, yielding lower quality evidence (Table 1). On the other hand, conducting prospective studies on this field can be very difficult, given the low prevalence of PJI after THA. Moreover, many series have determined certain independent risk factors, while others have failed to confirm the role of the same variables in increasing the infection risk (Table 1). One should be therefore very careful in interpreting the results of these studies. Further investigation with higher-quality trials is warranted, in order to formulate evidence-based guidelines for managing patients with risk factors for infection, scheduled to undergo THA.

## Figures and Tables

**Table 1:** Studies of risk factors for surgical site infection in patients undergoing total hip arthroplasty (THA).

Study	Type	Number of THAs	Risk factors identified
Berbari et al. [42]	Retrospective	526	Superficial surgical site infection, NNIS index score ≥1, malignancy, prior arthroplasty.

Bongartz et al. [37]	Retrospective	328	Revision surgery, prolonged duration of surgery, previous joint infection, and rheumatoid arthritis.

Bozic et al. [19]	Retrospective	40,919	Obesity, rheumatologic disease, coagulopathy, and preoperative anemia.

Bozic et al. [63]	Retrospective	57,047 THAs with different bearing surfaces	Metal-on-metal bearing surfaces.

Carroll et al. [17]	Retrospective	453	Obesity, allogeneic blood transfusion, coagulation with warfarin, and surgical skin preparation with 0.5% chlorhexidine in 70% alcohol.

Choong et al. [20]	Retrospective	819	Obesity and presence of >2 comorbidities.

Cordero-Ampuero and de Dios [8]	Retrospective	47	Older age, systemic corticosteroid treatment, prolonged duration of surgery, trauma, malignancy, liver disease, alcohol abuse, IV drug abuse, inadequate antibiotic prophylaxis, persistent wound secretion, dislocation, skin infection, urinary tract infection, abdominal infection, and pneumonia.

Dale et al. [11]	Retrospective	432,168	Trauma, male gender, hybrid fixation, cement without antibiotics, inflammatory arthritis, and avascular necrosis.

Dowsey and Choong [21]	Retrospective	1,207	Obesity.

Font-Vizcarra et al. [26]	Prospective	402	BMI ≥ 35.

Friedman et al. [22]	Retrospective	12,355	Obesity.

Geubbels et al. [69]	Prospective	13,608 THAs and hemiarthroplasties	Low annual institution volume.

Gilson et al. [39]	Retrospective	22 patients receiving TNFa blockers subjected to hip, knee, shoulder, and ankle arthroplasty	Treatment with TNFa blockers.

Smith et al. [53]	Prospective	308 THAs and TKAs	Allogeneic WBC-filtered blood transfusion.

Iorio et al. [35]	Retrospective	1,659	Diabetes.

Jafari et al. [45]	Retrospective	55 THAs and TKAs	Previous joint infection.

Jiang et al. [43]	Retrospective	878 THAs	Liver cirrhosis.

Katz et al. [70]	Retrospective	58,521	Low institution volume and low surgeon volume.

Lai et al. [31]	Retrospective	22	Diabetes and presence of >2 comorbidities.

Lee et al. [72]	Retrospective	74	Admission from a healthcare facility.

Malinzak et al. [9]	Retrospective	2,775	Younger age, diabetes, and obesity.

Maoz et al. [23]	Retrospective	3,672 primary THAs, 406 revision THAs	Obesity, revision surgery, prolonged duration of surgery, and non-same day surgery.

McDougall et al. [59]	Retrospective	1,047	Anticoagulation with warfarin or IV heparin.

Momohara et al. [38]	Retrospective	81	Treatment with TNFa blockers and longer duration of rheumatoid arthritis.

Mraovic et al. [24]	Retrospective	101 THAs and TKAs versus 1,847 controls	Diabetes, obesity, prolonged duration of surgery, presence of >2 comorbidities, history of myocardial infarction, congestive heart failure, and renal insufficiency.

Muilwijk et al. [71]	Retrospective	15,906	Low surgeon volume.

Namba et al. [14]	Retrospective	30,491	Obesity, female gender, ASA score ≥3, and bilateral THAs.

Newman et al. [55]	Retrospective	1,622	Allogeneic blood transfusion.

Ong et al. [12]	Retrospective	39,929	Prolonged duration of surgery, Charlson index >5, male gender, and lower socioeconomic status.

Parvizi et al. [60]	Retrospective	35	INR > 1.5.

Patel et al. [27]	Retrospective	1,221	Obesity, coagulation with LMWH, and increased drain tube loss.

Peel et al. [18]	Prospective	36	Obesity, superficial surgical site infection, increased drain tube loss, and systemic corticosteroid treatment.

Poultsides et al. [10]	Retrospective	412,356	Malignancy, coagulopathy, liver disease, male gender, congestive heart failure, fluid and electrolyte disorders, pulmonary circulatory disease, and minority race.

Pulido et al. [25]	Retrospective	5,060	Obesity, allogeneic blood transfusion, urinary tract infection, history of myocardial infarction, renal insufficiency, bilateral THAs, postoperative atrial fibrillation, and prolonged hospitalization.

Ridgeway et al. [6]	Prospective	16,291 primary THAs, 2,550 revision THAs	Older age, prolonged duration of surgery, trauma, and ASA score ≥3.

Saleh et al. [28]	Prospective	33 THAs and TKAs	Hematoma formation and persistent drainage.

Song et al. [34]	Retrospective	3,422	Diabetes, revision surgery, prolonged duration of surgery, and trauma.

Sousa et al. [49]	Prospective	1,248 THAs	Asymptomatic bacteriuria.

van Kasteren et al. [30]	Prospective	1,922	Prolonged duration of surgery, ASA score ≥3, and administration of prophylactic antibiotics after incision.

Willis-Owen et al. [13]	Prospective	1,750	Male gender and prolonged duration of surgery.

Wu et al. [7]	Retrospective	198	Older age, diabetes, obesity, alcohol abuse, and rural residence.
